# Variations in Flavonoid Metabolites Among *Forsythia suspensa* Populations in Response to Environmental Heterogeneity

**DOI:** 10.3390/plants14213329

**Published:** 2025-10-30

**Authors:** Shanshan Zhou, Longni Wu, Yahui Zhang, Yutong Guo, Jialan Xi, Danyang Li, Jinlan Ji

**Affiliations:** Faculty of Environmental Science and Engineering, Shanxi Institute of Science and Technology, Jincheng 048011, China

**Keywords:** *Forsythia suspensa*, flavonoid, metabolic profiling, environmental variables, environmental heterogeneity

## Abstract

*Forsythia suspensa* (Thunb.) Vahl, a pharmacopoeial medicinal plant, is valued for its therapeutic efficacy in heat-clearing detoxification, dispelling wind-heat, and promoting blood circulation to resolve stasis. Flavonoids, ubiquitous secondary metabolites in *F. suspensa*, are critically linked to pharmacological activities and exhibit diverse biological functions. To elucidate the chemotypic divergence and ecological drivers of its bioactive compounds, we conducted flavonoid metabolomic profiling across ten wild populations *F. suspensa* using UPLC-MS/MS. Results revealed significant inter-population variation in all twenty-nine flavonoid metabolites analyzed. Notably, Notably, Cinchonain Ic was significantly enriched in the JX population, Flavanomarein in the LT population, and Desmethylxanthohumol in the HX population. Association analysis with environmental variables further indicated that Sulfuretin, Apigenin-5-O-glucoside, and Flavanomarein were positively correlated with multiple precipitation-related variables (bio12-Annual Precipitation, bio14-Precipitation of Driest Month, bio17-Precipitation of Driest Month, and bio19-Precipitation of Coldest Quarter), whereas Vicenin 2 was negatively correlated with bio12, bio17, and bio19. Homoplantaginin showed a positive correlation with bio4 (Temperature Seasonality) and bio7 (Temperature Annual Range). Loureirin B was positively correlated with elevation but negatively correlated with high-temperature variables (bio5-Max Temperature of Warmest Month, bio8-Mean Temperature of Wettest Quarter, and bio10-Mean Temperature of Warmest Quarter). 5-Demethoxynobiletin was positively associated with both precipitation (bio12, bio17, bio19) and temperature variables (bio1-Annual Mean Temperature, bio6-Min Temperature of Coldest Month, bio9-Mean Temperature of Driest Quarter and bio11-Mean Temperature of Coldest Quarter). Cinchonain Ic was positively correlated with bio2 (Mean Monthly Temperature Range), and Oroxin A was negatively correlated with elevation. These findings demonstrated that flavonoids accumulation in *F. suspensa* was predominantly influenced by temperature heterogeneity, with precipitation serving as a secondary factor, while latitude and elevation play only limited roles. This study systematically investigates the divergence and environmental drivers of flavonoids in *F. suspensa* populations, clarifies the molecular ecological basis of its adaptation to environmental heterogeneity, and provides valuable insights for leveraging ecological factors to enhance medicinal potential, ultimately supporting targeted breeding and optimized field management strategies.

## 1. Introduction

*Forsythia suspensa* (Thunb.) Vahl (Oleaceae) is a widely used bulk medicinal herb whose fruit, *Forsythiae Fructus*, has been pharmacologically documented since the *Shen Nong Ben Cao Jing* [1]. It is currently recognized in the pharmacopeias of China, Japan, the United States, and South Korea [2,3,4,5]. The greenish fruits that start to ripen are known as Qingqiao, whereas the fully ripened yellow fruits are collected as Laoqiao [1,6]. Characterized by a bitter taste, subtle fragrance, and cold property, this herb acts on the lung, heart, and small intestine meridians [1,6]. Its demonstrated bioactivities include heat-clearing, detoxification, nodule-dispersion, and wind-heat dispersion, with clinical applications spanning fever, inflammatory conditions, gonorrhea, and early-stage warm pathogen diseases [6]. Revered as the “holy herb for ulcerative disorders”, it holds significant medicinal, ecological, and economic value.

Plant growth is influenced by environmental factors, to which plants respond through two principal strategies: adjustments in distribution and the generation of genomic adaptive variations [7,8]. Environmental changes are primarily reflected in climatic fluctuations over temporal scales and environmental heterogeneity across spatial scales [9]. Environmental heterogeneity—defined by spatiotemporal variation in abiotic (e.g., temperature, moisture, solar radiation, and edaphic factors) and biotic conditions—plays a critical role in modulating plant secondary metabolism. Abiotic stressors such as drought, thermal extremes, and UV-B radiation can induce adaptive responses that alter metabolite biosynthesis, including flavonoid metabolites [10,11,12]. *F. suspensa* produces a wide array of structurally diverse bioactive metabolites, including flavonoids, lignans, phenylethanoid glycosides, and terpenoids [1,13,14]. Among these, flavonoids are of particular pharmacological importance due to their broad spectrum of bioactivities [15]. Flavonoids contribute significantly to plant adaptation to environmental stresses, enhancing resistance to abiotic challenges such as drought, extreme temperatures, high salinity, and UV exposure [16,17]. Furthermore, they play essential roles in various physiological and developmental processes, and influence the agronomic and industrial quality of plant-derived products [18,19]. Moreover, flavonoids exhibite a broad spectrum of health-promoting properties, including anti-inflammatory, antifungal, antimicrobial, antiviral, antioxidant, and antitumor activities [20]. They aid in scavenging free radicals, stabilizing reactive oxygen species, and protecting against chronic diseases such as cancer, diabetes, and cardiovascular disorders [20,21]. Additionally, they contribute to immune modulation by suppressing pro-inflammatory responses, especially those involving auto-reactive T cells [20,22].

The main source of medicinal materials for *F. suspensa* is derived from wild resources. *F. suspensa* in China is widely distributed across mountainous regions (altitude: 250–2200 m), primarily in Shanxi, Henan, Shaanxi, and Hebei provinces. Variations in growing environments and climates are the primary reasons for the significant differences in the content of bioactive metabolites among *F. suspensa* populations [9,23,24,25]. Given the extensive distribution and diversity of wild *F. suspensa* resources, along with the considerable variation in the content of medicinal active substances and the tendency for varietal changes during cultivation, the germplasm resources and quality of *F. suspensa* have become the main constraints on the development of the industry and its pharmaceutical applications. Therefore, selecting high-quality varieties is one of the key issues in the *F. suspensa* cultivation industry.

Previous research on *F. suspensa* has predominantly focused on its chemical composition, pharmacological properties, and quantitative analysis [6,13]. Pharmacological investigations have revealed that *F. suspensa* exhibits notable anti-inflammatory, antimicrobial, and anticancer activities. Guo et al. suggested that these properties contribute to its traditional use in clearing heat and detoxification [26]. Furthermore, Wang et al. reported that its antioxidant and anti-inflammatory characteristics hold promising therapeutic potential for cognitive impairment and Parkinson’s disease [27]. From a biosynthetic perspective, integrated genomic and transcriptomic analyses by Li et al. proposed multiple biosynthetic pathways for forsythiaside and forsythiaside A, identifying 48 candidate genes involved in their synthesis [28]. In addition to its chemical and pharmacological profile, environmental factors have been shown to significantly influence the growth and quality of *F. suspensa*. Studies indicated that altitude and slope orientation critically affect both the yield and quality of cultivated plants [29,30]. Fu et al. demonstrated a strong correlation between geographic distance and population genetic differentiation [24]. Suo et al. identified precipitation as a key factor influencing growth [31], while Li et al. highlighted the impact of temperature on its geographical distribution [25]. Although previous studies have addressed environmental impacts on the growth and distribution of *F. suspensa*, the effect of such variations on the content of flavonoid metabolites remains largely unexplored. It is therefore of significant interest to investigate whether environmental changes also alter the content distribution of flavonoids in *F. suspensa*. To elucidate the environmental effects on flavonoid metabolism, we performed UPLC-MS/MS-based metabolic profiling of flavonoids across 10 populations of *F. suspensa*, integrating environmental data to identify the key factors governing their accumulation. This study could not only shed light on the molecular ecological basis for *F. suspensa* adapted to environmental heterogeneity but also provide important insights into how ecological factors shape the medicinal potential of this species, ultimately informing targeted breeding and optimized field management.

## 2. Results

### 2.1. Variation in Flavonoid Metabolites Among F. suspensa Populations

**Figure 1:**
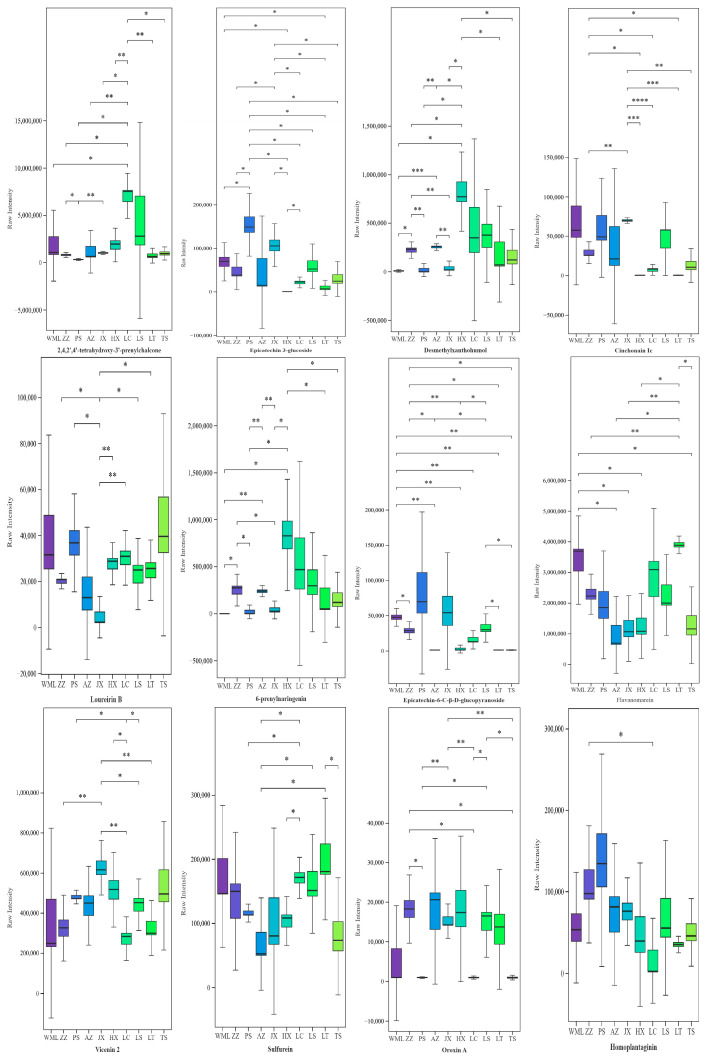
Advanced significance box plot of 12 flavonoid metabolites in *F. suspensa*. Asterisks indicated significant differences according to Student’s *t*-test (*, *p* < 0.05; **, *p* <  0.01; ***, *p* < 0.001; ****, *p* < 0.0001).

All twenty-nine flavonoid metabolites we identified showed significant inter-population variation across 10 *F. suspensa* populations based on the one-way ANOVA (Figure S1). Differences in metabolite levels between the two groups were visualized using box plots based on Student’s *t*-test (Figure 1 and Figure S2). As shown in Figure 1, multiple metabolites displayed distinct population-specific accumulation patterns: 2,4,2′,4′-tetrahydroxy-3′-prenylchalcone was significantly higher in the LC population than in the other eight populations; Epicatechin 3-glucoside in PS and Desmethylxanthohumol in HX were significantly higher than in the other seven populations; Epicatechin-6-C-*β*-*D*-glucopyranoside in WML was significantly higher than in the other six populations; Epicatechin 3-glucoside in JX, Cinchonain Ic in JX, 6-prenylnaringenin in HX, and Flavanomarein in LT were significantly higher than in the other five populations.

**Figure 2:**
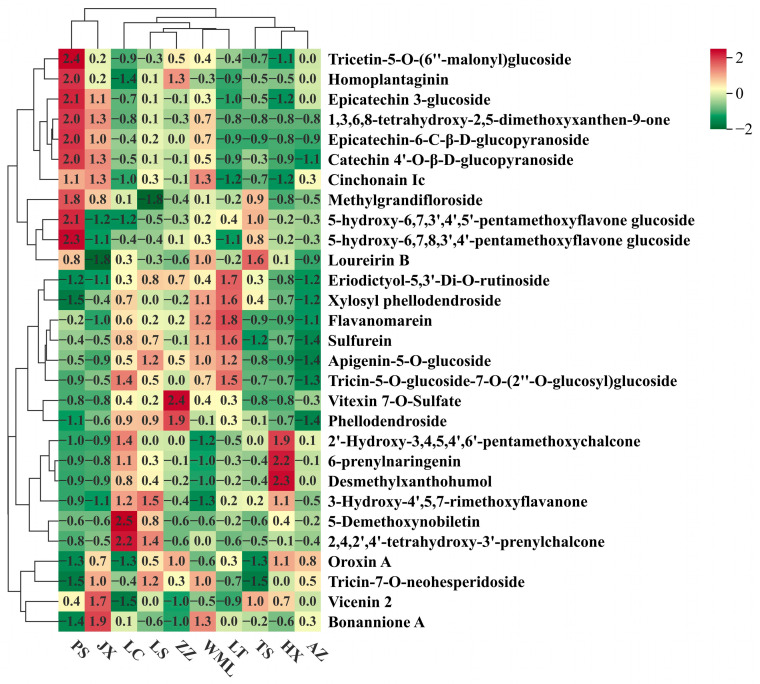
Cluster heatmap of flavonoid metabolites in *F. suspensa*. Colour depth represents the average intensity of metabolite contents. Red represents the highest content, green represents the lowest content.

**Figure 3:**
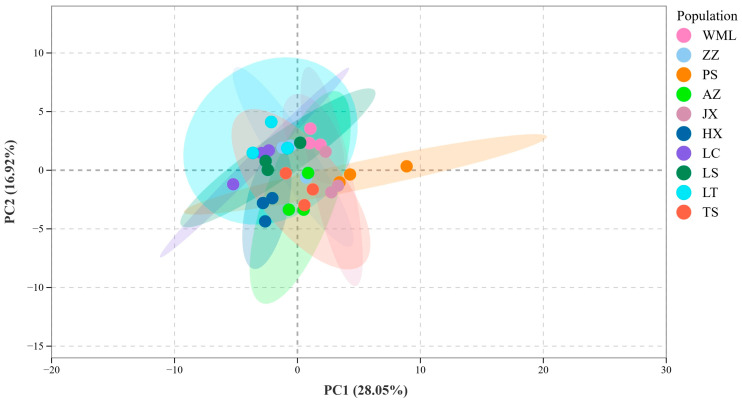
PCA score plot of flavonoid metabolites in *F. suspensa*. The ellipses represent 95% confidence ellipses for each group.

Having a comprehensive view of flavonoid profile similarities and differences among *F. suspensa* populations, cluster heatmap (Figure 2) and PCA (Figure 3) were performed. Hierarchical clustering grouped the ten populations into three main clusters (Figure 2). Integrating these results with the significance testing in Figure 1 revealed the following patterns: Cluster 1 contained populations PS and JX. PS showed high accumulation of ten metabolites (Figure 2), though only Epicatechin 3-glucoside reached statistical significance (Figure 1). JX exhibited moderate accumulation of eight metabolites, with only Epicatechin 3-glucoside and Cinchonain Ic being statistically significant. Cluster 2 (LC, LS, ZZ, WML and LT) displayed moderate levels of certain metabolites with clear subclustering, where 2,4,2′,4′-tetrahydroxy-3′-prenylchalcone was significantly enriched in LC, Epicatechin-6-C-*β*-*D*-glucopyranoside in WML, and Flavanomarein in LT. Cluster 3 (TS, HX, and AZ) generally showed low flavonoid levels, with only Desmethylxanthohumol and 6-prenylnaringenin significantly enriched in HX.

PCA further confirmed distinct metabolic differentiation among *F. suspensa* populations (Figure 3). The first two principal components, Principal Component 1 (PC1, 28.05%) and 2 (PC2, 16.92%), collectively explained 44.97% of the total metabolic variance among samples. The score plot showed clear separation among all populations, reflecting substantial inter-population metabolic diversity. Population clustering within the PCA ordination demonstrated metabolic similarity: PS and JX formed a close group, indicating similar flavonoid profiles; TS, HX, and AZ clustered together; and LC, LS, ZZ, WML, and LT comprised a third cluster. These patterns were consistent with the hierarchical clustering results (Figure 2), confirming robust population-level differentiation in flavonoid composition across the studied *F. suspensa* populations.

### 2.2. Relationship Between Environmental Variables and Flavonoid Metabolites in F. suspensa

**Figure 4:**
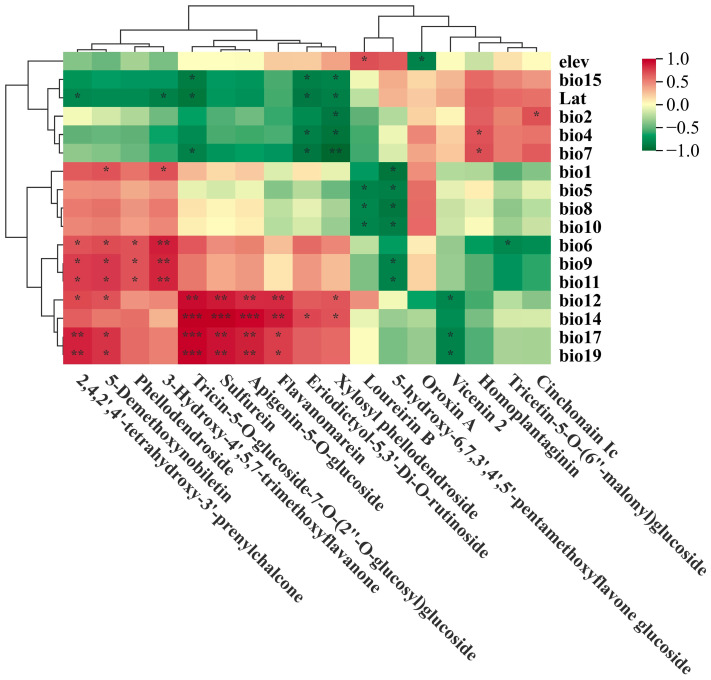
Cluster heatmap based on correlation between flavonoid metabolites and the environmental variables. Red represents positive correlation, green represents negative correlation. Asterisks indicated statistical significant (*, *p* < 0.05; **, *p* < 0.01; ***, *p* < 0.001).

A correlation cluster heatmap was implemented to identify the major environmental variables governing the flavonoid metabolites in *F. suspensa*. Figure 4 presented significant correlations (*p*-values < 0.05). We discovered that temperature notably impacted 12 metabolites, precipitation significantly affected 9 flavonoid metabolites, latitude influenced 5 metabolites, and elevation affected 2 metabolites (Figure 4).

#### 2.2.1. Temperature-Correlated Metabolites

Six flavonoid metabolites exhibited significant positive correlations with temperature-related variables: 2,4,2′,4′-tetrahydroxy-3′-prenylchalcone and Phellodendroside were correlated with bio6 (Min Temperature of Coldest Month), bio9 (Mean Temperature of Driest Quarter) and bio11 (Mean Temperature of Coldest Quarter); 5-Demethoxynobiletin and 3-Hydroxy-4′,5,7-trimethoxyflavanone with bio1 (Annual Mean Temperature), bio6, bio9 and bio11; Homoplantaginin with bio4 (Temperature Seasonality) and bio7 (Temperature Annual Range); Cinchonain Ic with bio2 (Mean Monthly Temperature Range).

Six metabolites exhibited significant negative correlations with temperature-related variables: Tricin-5-*O*-glucoside-7-*O*-(2″-*O*-glucosyl)glucoside with bio7; Eriodictyol-5,3′-*Di*-*O*-rutinoside with bio4 and bio7; Xylosyl phellodendroside with bio2, bio4 and bio7; Loureirin B with bio5 (Max Temperature of Warmest Month), bio8 (Mean Temperature of Wettest Quarter), bio10 (Mean Temperature of Warmest Quarter); 5-hydroxy-6,7,3′,4′,5′-pentamethoxyflavone glucoside with bio1, bio5, bio8, bio9, bio10, bio11); Tricetin-5-*O*-(6″-malonyl)glucoside with bio6.

#### 2.2.2. Precipitation-Correlated Metabolites

Eight metabolites showed significant positive correlation with precipitation-related variables: 2,4,2′,4′-tetrahydroxy-3′-prenylchalcone and 5-Demethoxynobiletin were correlated with bio12 (Annual Precipitation), bio17 (Precipitation of Driest Quarter), and bio19 (Precipitation of Coldest Quarter); Tricin-5-*O*-glucoside-7-*O*-(2″-*O*-glucosyl) glucoside, Sulfurein, Apigenin-5-*O*-glucoside and Flavanomarein with bio12, bio14 (Precipitation of Driest Month), bio17, and bio19; Eriodictyol-5,3′-*Di*-*O*-rutinoside with bio14; Xylosyl phellodendroside with precipitation bio12 and bio14.

Four metabolites exhibited significant negative correlations with precipitation-related variables: Tricin-5-*O*-glucoside-7-*O*-(2″-*O*-glucosyl)glucoside, Eriodictyol-5,3′-*Di*-*O*-rutinoside, and Xylosyl phellodendroside with bio15 (Precipitation Seasonality); Vicenin 2 with precipitation bio12, bio17 and bio19.

#### 2.2.3. Latitude and Elevation Correlated Metabolites

Latitude revealed a significant negative correlation with five metabolites: 2,4,2′,4′-tetrahydroxy-3′-prenylchalcone, 3-Hydroxy-4′,5,7-trimethoxyflavanone, Eriodictyol-5,3′-*Di*-*O*-rutinoside, Xylosyl phellodendroside, and Tricin-5-*O*-glucoside-7-*O*-(2″-*O*-glucosyl)glucoside. In contrast, elevation was positively correlated with Loureirin B but negatively correlated with Oroxin A.

## 3. Discussion

The observed variation in flavonoid metabolites among *F. suspensa* populations underscores the significant influence of environmental heterogeneity on its phytochemical profile. As a plant traditionally revered as the “holy herb of the ulcer family” and highly valued in Traditional Chinese Medicine for its pharmacological, ecological, and economic benefits [5], these findings provide important insights into how ecological factors shape the medicinal potential of this species

Our study revealed that multiple flavonoids exhibited population-specific enrichment patterns coupled with defined environmental correlations, underscoring their ecological and medicinal potential relevance. For instance, Vicenin 2 has been reported to exhibit a range of promising biological activities, including antidiabetic, antioxidant, anti-inflammatory, and anticancer effects, as well as the ability to promote cell proliferation and migration [32]. In our study, Vicenin 2 was significantly negatively correlated with precipitation-related variables (bio12, bio17, bio19). Flavanomarein exhibits diverse pharmacological activities, including potent antioxidant effects through free radical scavenging and lipid peroxidation inhibition, as well as antidiabetic, antihypertensive, and anti-hyperlipidemic properties [33]. In our study, Flavanomarein was significantly enriched in the LT population and positively correlated with precipitation-related variables (bio12, bio14, bio17, bio19). Similarly, Sulfuretin, with a broad spectrum of activities including neuroprotective, antioxidant, anticancer, hepatoprotective, antimicrobial, anti-inflammatory, antidiabetic, and anti-obesity effects [34], also showed positive correlations with precipitation-related variables (bio12, bio14, bio17, bio19) in our study. And Apigenin-5-*O*-glucoside, known for its antioxidant properties and potential in pharmaceutical and cosmetic applications [35], was positively correlated with precipitation(bio12, bio14, bio17, and bio19). Desmethylxanthohumol, which induces apoptosis, shows antiproliferative and antioxidant activities, and acts as a chemoprotective agent [36], was enriched in the HX population in our study. Homoplantaginin has long been utilized in the treatment of inflammatory conditions, including hepatitis, cough, and diarrhea. It exhibits notable anti-inflammatory and antioxidant properties, effectively inhibiting the expression of pro-inflammatory cytokines. These mechanisms contribute to its protective effects against lipopolysaccharide-induced hepatic injury, as well as inflammation triggered by lipids and high glucose in endothelial cells [37]. In our study, Homoplantaginin was significantly positively correlated with temperature-related variables (bio4, bio7). Loureirin B, displaying antibacterial, anti-inflammatory, immunomodulatory, insulin-sensitizing, and neuroprotective activities [38], was positively correlated with elevation and negatively with temperature (bio5, bio8, bio10) in our study. 5-Demethoxynobiletin, with anti-inflammatory, pro-apoptotic, pro-autophagic, and melanogenesis-inducing effects [39], was positively correlated with precipitation (bio12, bio17, bio19) and temperature (bio1, bio6, bio9, bio11). Cinchonain Ic belongs to the cinchonains, which are a class of naturally occurring flavonolignans exhibiting diverse biological activities spanning cytotoxic, antioxidant, anti-inflammatory, antimicrobial, neuroprotective, antidiabetic, and anticancer effects [40]. In our study, Cinchonain Ic was significantly enriched in the JX population and positively correlated with Mean Monthly Temperature Range (bio2). Oroxin A, which has antioxidant, anti-breast cancer, and antidiabetic properties [41], was negatively correlated with elevation in our study. Together, these metabolite-specific environmental responses illustrated how environmental heterogeneity regulates the flavonoid metabolites of *F. suspensa* and thus affects its medicinal potential.

Temperature emerged as the principal abiotic factor shaping flavonoid accumulation in our study. Low temperature is a primary limiting factor for *F. suspensa* cultivation, restricting both suitable planting regions and overall yield [42]. Recent research on cold resistance indicates that *F. suspensa* employs adaptive strategies, such as increased accumulation of metabolites to enhance osmotic potential and mitigate cold damage [43]. This is consistent with evidence of cold adaptive differentiation among natural populations [25], differential expression in cold-tolerance pathways [44], and genetic divergence driven by Pleistocene climate fluctuations [23]. Mantel tests and redundancy analysis further link population genetic differentiation to geographical distance, temperature, and latitude [24], consistent with our finding that temperature governs flavonoid-based adaptation. While some studies showed that low temperature would increase the accumulation of flavonoids (e.g., quercetin and rutin) [45,46], others indicate elevated temperature can also enhance flavonoid content [47]. Our correlation analyses clearly established temperature as the principal abiotic factor sculpting flavonoid profiles. Six metabolites (2,4,2′,4′-tetrahydroxy-3′-prenylchalcone, 5-Demethoxynobiletin, Phellodendroside, 3-Hydroxy-4′,5,7-trimethoxyflavanone, Homoplantaginin, and Cinchonain Ic) showed strong positive correlations with temperature, while Six others (Tricin-5-*O*-glucoside-7-*O*-(2″-*O*-glucosyl)glucoside, Eriodictyol-5,3′-*Di*-*O*-rutinoside, Xylosyl phellodendroside, Loureirin B, 5-hydroxy-6,7,3′,4′,5′-pentamethoxyflavone glucoside, and Tricetin-5-*O*-(6″-malonyl)glucoside) exhibited negative correlations (Figure 4), indicating complex regulatory mechanisms underlying flavonoid biosynthesis.

Precipitation emerged as a secondary influential factor, significantly affecting nine metabolites in our study. Recent research has reported strong correlations between flavonoid metabolites and latitude, longitude, and precipitation gradients [10]. In our study, latitude was negatively correlated with five metabolites (2,4,2′,4′-tetrahydroxy-3′-prenylchalcone, 3-Hydroxy-4′,5,7-trimethoxyflavanone, Eriodictyol-5,3′-*Di*-*O*-rutinoside, Xylosyl phellodendroside, and Tricin-5-*O*-glucoside-7-*O*-(2″-*O*-glucosyl)glucoside). Although some studies noted positive correlations between quercetin derivatives and latitude [10,48,49], and others reported increased flavonoid content with elevation [50,51,52], we found that elevation was positively correlated with Loureirin B and negatively correlated with Oroxin A. Population genomic studies in *F. suspensa* support these environment-metabolite associations, having identified candidate genes for local adaptation related to solar radiation, temperature, and water availability [25], and revealing genetically distinct populations shaped by divergent selection in response to multiple environmental variables [9]. Our results deepen this understanding by clarifying specific relationships between precipitation, temperature, and the abundance of individual flavonoid in *F. suspensa*.

In summary, this study demonstrated significant inter-population variation in flavonoid metabolites in *F. suspensa*, which was primarily driven by temperature heterogeneity, with precipitation as a secondary factor, while latitude and elevation played only a minimal role. By decoding key environment-metabolite relationships, we provide a framework for climate-smart cultivation, germplasm conservation, and metabolite-directed breeding. These insights bridge ecological pharmacology with industrial application, facilitating the sustainable production of this valuable medicinal resource under global change.

These results further illuminate how ecological niche differentiation shapes adaptive divergence and offer valuable genetic resources for Forsythia varietal development. However, we acknowledge certain limitations, including restricted population sampling, lack of soil physicochemical analysis, and insufficient seasonal or molecular-level data—all of which may influence the interpretation of metabolite variation. Given that soil properties (e.g., pH, nutrient availability, and organic matter content) interact strongly with climatic factors in shaping secondary metabolism, and considering the complexity of flavonoid biosynthesis, future work should integrate soil analysis with multi-omics approaches—particularly metabolomics and transcriptomics—to more comprehensively elucidate how environmental variables regulate rate-limiting steps in the flavonoid biosynthetic pathway.

## 4. Materials and Methods

### 4.1. Plant Materials

**Figure 5:**
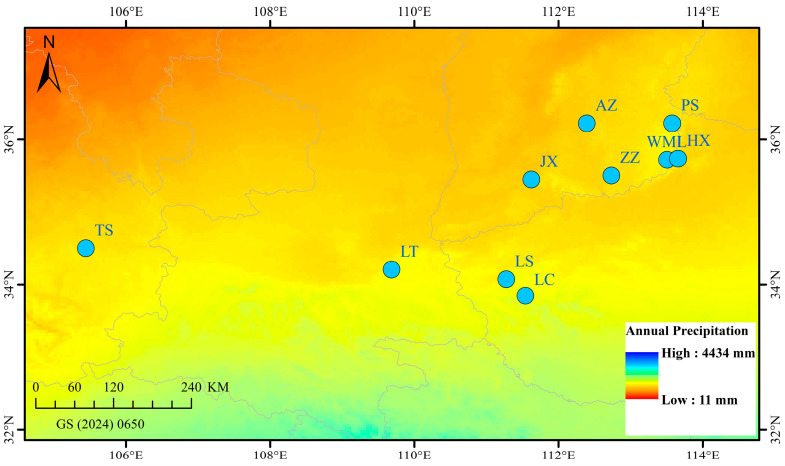
Geographical distribution of 10 *F. suspensa* populations in China.

From late July to early August 2024, fruit samples (Qingqiao) were collected from 10 wild *F. suspensa* populations spaced at least 30 km apart (Figure 5 and Table 1). Three samples were randomly collected from each population, with an inter-individual spacing of at least 50 m. The latitude and longitude of each population were precisely recorded using GPS (Table 1). The collected samples were immediately frozen in liquid nitrogen and stored at −80 °C for future use.

### 4.2. Flavonoids Metabolic Profiling

The samples of *F. suspensa* were vacuum freeze-dried for 63 h in a freeze dryer (Scientz-100F). For each sample, 50 mg of homogenized sample powder was suspended in 1200 µL-20 °C pre-cooled 70% methanol aqueous internal standard extraction solution. The internal standard extraction solution was prepared by dissolving 1 mg of the standard (2-Chlorophenylalanine) in 1 mL of 70% methanol to make a 1000 µg/mL stock solution, which was further diluted with 70% methanol to prepare a 250 µg/mL internal standard solution. The mixture was vortexed (Vortex Mixer, VORTEX-5, Kyllin-Bell, Haimen, China)) every 30 min for 30 s each time, totaling 6 times. After centrifugation (Centrifuge, 5424R, Eppendorf, Hamburg, Germany) at 11,304 g for 3 min, the supernatant was collected, filtered through a microporous membrane (0.22 µm pore size) in preparation for UPLC-MS/MS analysis. The water used was ultrapure, while methanol, formic acid, and acetonitrile were all of chromatographic grade.

Analysis of flavonoid metabolites using Ultra-Performance Liquid Chromatography (UPLC, ExionLC™ AD, SCIEX, Fremont, CA, USA) Coupled with Tandem Mass Spectrometry (MS/MS) at Metware Biotechnology Co., Ltd. (Wuhan, China).

As the non-targeted approach provides the advantage of discovering important metabolites that might otherwise remain undetected with a targeted approach, to identify the key metabolites and metabolic pathways, particularly the major secondary metabolic pathways, involved in plateau adaptation for sandrice, samples with three biological replicates of each ecotype were prepared for UPLC-MS non-targeted metabonomics using LC-ESI-Q TRAP-MS/MS systemsChromatographic conditions mainly included: (1) Column: Agilent SB-C18 1.8 µm, 2.1 mm × 100 mm; (2) Mobile phase: phase A was ultrapure water with 0.1% formic acid, phase B was acetonitrile containing 0.1% formic acid; (3) Elution gradient: At 0.00 min, mobile phase B was maintained at 5%; then linearly increased to 95% over 9.00 min and held at 95% for 1 min (9.00–10.00 min); subsequently, the phase B was rapidly reduced to 5% from 10.00 to 11.10 min, followed by a re-equilibration period at 5% until 14.00 min. (4) Flow rate: 0.35 mL/min; Column Temperature: 40 °C; Injection Volume: 2 µL. The effluent was alternatively connected to an ESI-triple quadrupole-linear ion trap (QTRAP)-MS.

Mass spectrometric conditions mainly included: electrospray ionization (ESI) source temperature was maintained at 500 °C with ion spray voltages (IS) set at 5500 V in positive ion mode and −4500 V in negative ion mode. Optimized ion source gas parameters included ion source gas I (GSI) at 50 psi, GSII at 60 psi, and air curtain (CUR) at 25 psi, with collision-induced ionization set to high. The triple quadrupole (QQQ) mass spectrometer was operated in multi-reaction monitoring (MRM) mode using nitrogen as collision gas at medium pressure. To ensure accurate and reliable results, the declustering potential (DP) and collision energy (CE) were systematically optimized to determine the optimal parameters for each MRM ion transition.

Metabolites were annotated by mapping them to the self-built database MWDB (Metware Biotechnology Co., Ltd., Wuhan, China) to identify their chemical structures. The original peak areas obtained from the detection were used as the relative content of flavonoid metabolites in *F. suspensa* (Table S1). Although the absolute content of the substances could not be quantified, the consistent detection conditions allowed for comparative analysis of the same compound across different samples.

### 4.3. Environmental Variables

To test the correlations of different metabolites with environmental variables, we extracted 20 environmental variables (Table 2 and Table S2) of 10 *F. suspensa* populations under historical conditions (1970–2000) from the world climate database (https://www.worldclim.org/data/worldclim21.html, accessed on 15 February 2025), with 2.5 arcmin resolution.

### 4.4. Statistical Analyses

We performed metabolomic analysis using Metware Cloud platform, which integrated multiple analytical modules: (1) “Violin Plots” were applied to assess inter-population distribution patterns of metabolites across 10 *F. suspensa* populations based on one-way analysis of variance (ANOVA); (2) “Advanced significance box plot” were used to visualize differences between two groups using Student’s *t*-test; (3) “Cluster Heatmap” enabled population clustering based on metabolite profiles; (4) “Principal component analysis (PCA)” provided an overview of metabolic trends and overall variation in flavonoids; (5) “Correlation cluster heatmap” were employed to evaluate relationships between metabolite levels and environmental variables using Pearson Correlation.

## 5. Conclusions

This study revealed substantial variation in the content of twenty-nine flavonoid metabolites across ten *F. suspensa* populations. Temperature emerged as the dominant environmental modulator, significantly influencing twelve metabolites, followed by precipitation, which affected nine flavonoid metabolites. In contrast, latitude and elevation exhibited selective effects on specific metabolite subsets. These findings provide critical insights for guiding future metabolite-targeted breeding programs and cultivation management strategies for *F. suspensa*.

## Figures and Tables

**Table 1:** Details of the locality information of 10 wild *F. suspensa* populations.

Code	Location	Population Code	Longitude (N°)	Latitude (N°)
1	Wangmangling, Shanxi	WML	113°30′20″	35°43′9″
2	Zezhou, Shanxi	ZZ	112°44′3″	35°30′7″
3	Pingshun, Shanxi	PS	113°34′36″	36°13′18″
4	Anze, Shanxi	AZ	112°23′33″	36°13′13″
5	Jiangxian, Henan	JX	111°37′25″	35°26′50″
6	Huixian, Henan	HX	113°39′26″	35°44′8″
7	Luanchuan, Henan	LC	111°32′30″	33°50′47″
8	Lushi, Henan	LS	111°16′32″	34°4′21″
9	Lantian, Shaanxi	LT	109°41′4″	34°12′27″
10	Tianshui, Gansu	TS	105°26′35″	34°29′59″

**Table 2:** Content information of 22 environmental variables.

Abbreviation	Environmental Variables	Unit
bio1	Annual Mean Temperature	°C
bio2	Mean Monthly Temperature Range	°C
bio3	Isothermality (MMTR/TAR) (×100)	-
bio4	Temperature Seasonality (standard deviation ×100)	-
bio5	Max Temperature of Warmest Month	°C
bio6	Min Temperature of Coldest Month	°C
bio7	Temperature Annual Range	-
bio8	Mean Temperature of Wettest Quarter	°C
bio9	Mean Temperature of Driest Quarter	°C
bio10	Mean Temperature of Warmest Quarter	°C
bio11	Mean Temperature of Coldest Quarter	°C
bio12	Annual Precipitation	mm
bio13	Precipitation of Wettest Month	mm
bio14	Precipitation of Driest Month	mm
bio15	Precipitation Seasonality (Coefficient of Variation)	-
bio16	Precipitation of Wettest Quarter	mm
bio17	Precipitation of Driest Quarter	mm
bio18	Precipitation of Warmest Quarter	mm
bio19	Precipitation of Coldest Quarter	mm
elev	Elevation	m
Lon	Longitude	-
Lat	Latitude	-

## Data Availability

All data in this study can be found in the manuscript.

## Supplementary Materials

The following supporting information can be downloaded at: https://www.mdpi.com/article/10.3390/plants14213329/s1, Figure S1. Violin plot of 29 flavonoid metabolites in *F. suspensa*; Figure S2. Advanced significance box plot of 29 flavonoid metabolites in *F. suspensa*; Table S1. The relative content of 29 flavonoid metabolites in 10 *F. suspensa* populations; Table S2. Environmental variables of 10 *F. suspensa* populations from the world climate database (www.worldclim.org, accessed on 15 February 2025).
