# Supplementary material for: Variations in Flavonoid Metabolites Among Forsythia suspensa Populations in Response to Environmental Heterogeneity
Source: Plants (Basel). 2025 Oct 30;14(21):3329. doi: 10.3390/plants14213329 (PMC12610222; doi:10.3390/plants14213329)
Supplement: Supplementary file 1 [file plants-14-03329-s001.zip › Figure S1.pdf]

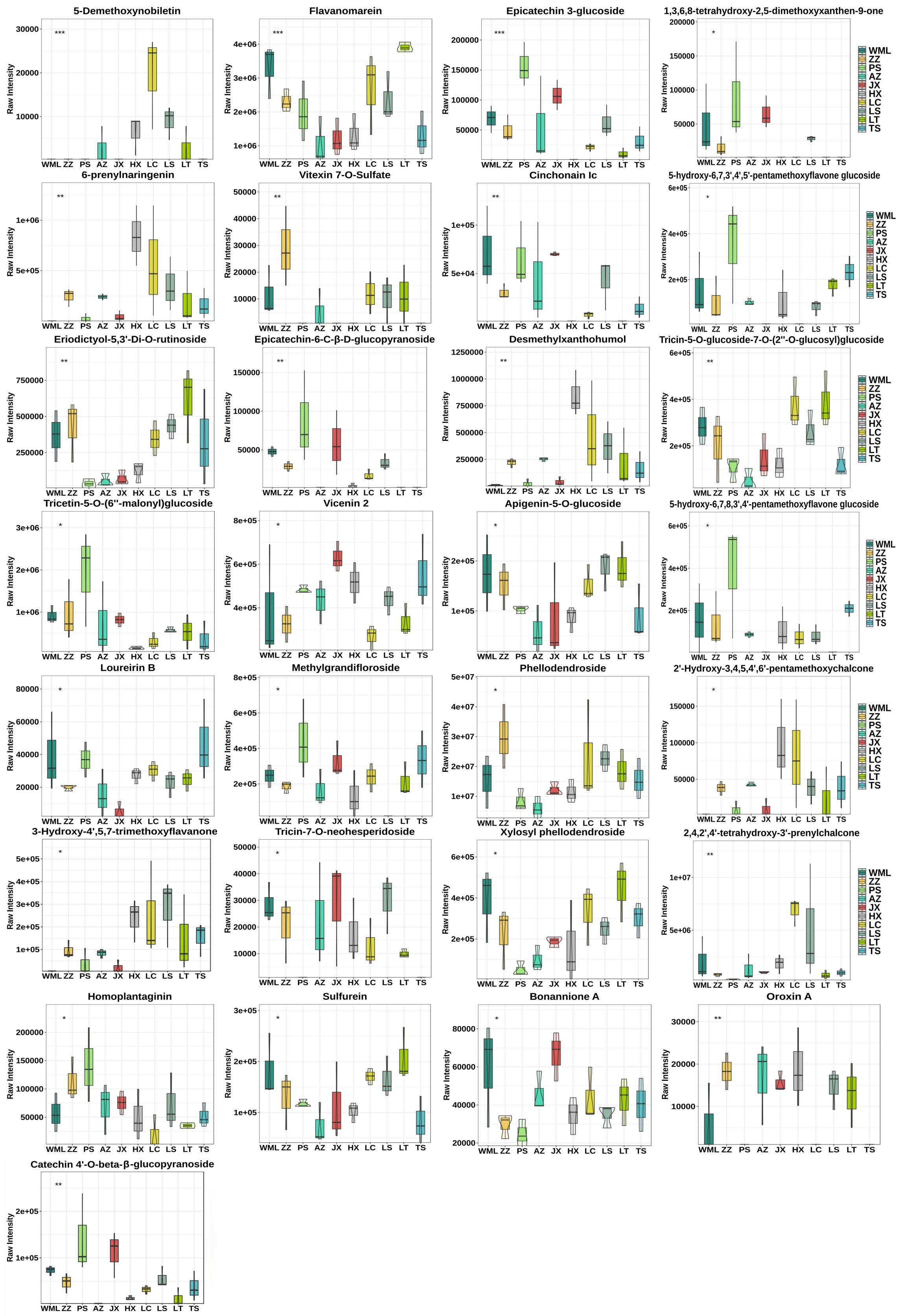

Figure S1 Violin plot of 29 flavonoid metabolites in *F. suspensa*.

Asterisks indicated significant differences according to ANOVA (*, p<0.05; **, P < 0.01; ***, p<0.001; ****, P < 0.0001).
