# Supplementary material for: Variations in Flavonoid Metabolites Among Forsythia suspensa Populations in Response to Environmental Heterogeneity
Source: Plants (Basel). 2025 Oct 30;14(21):3329. doi: 10.3390/plants14213329 (PMC12610222; doi:10.3390/plants14213329)
Supplement: Supplementary file 1 [file plants-14-03329-s001.zip › Figure S2.pdf]

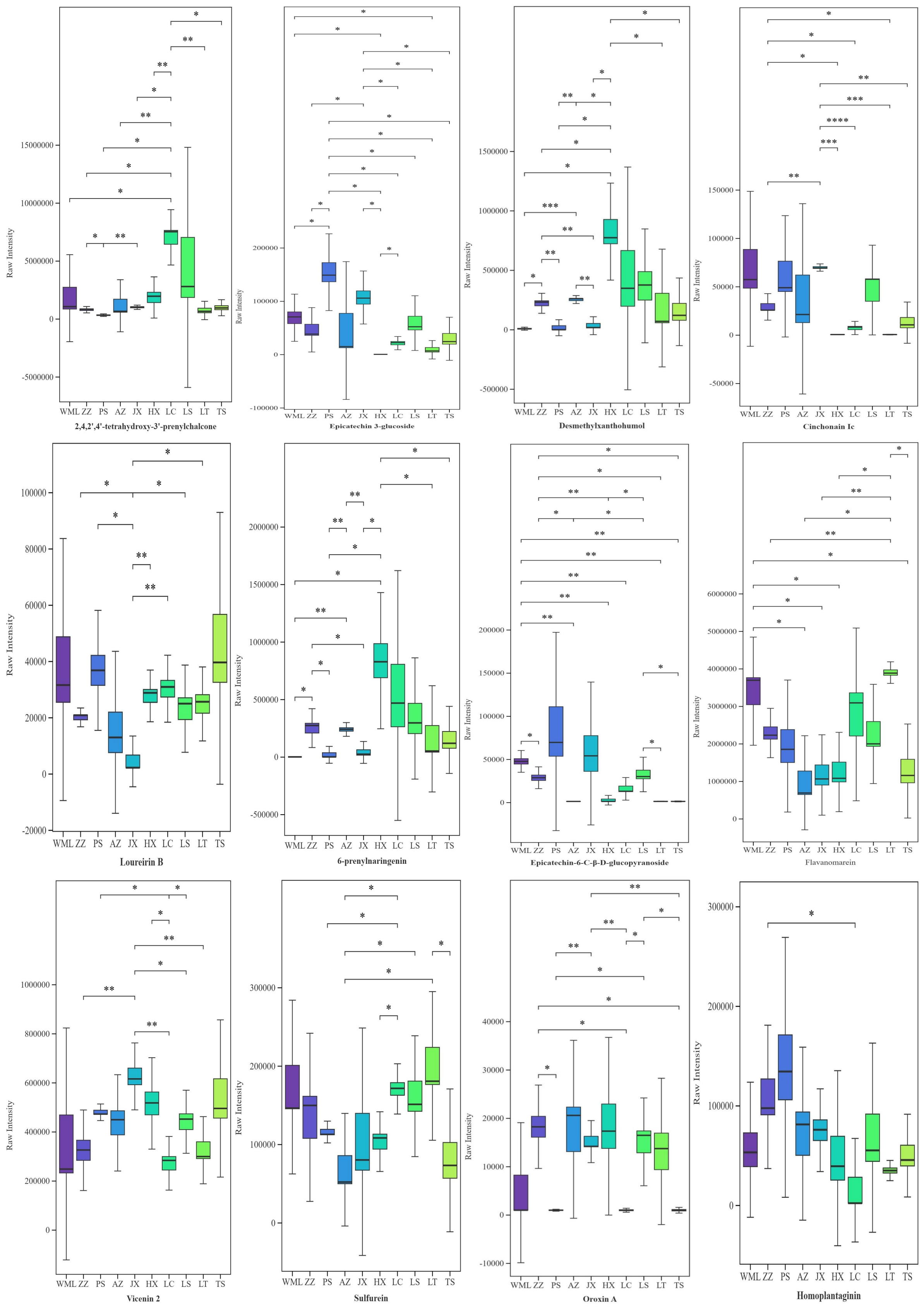
Raw Intensity
WML ZZ PS AZ JX HX LC LS LT TS
2,4,2',4'-tetrahydroxy-3'-prenylchalcone
Epicatechin 3-glucoside
Desmethylxanthohumol
Cinchonain Ic
Loureirin B
6-prenylnaringenin
Epicatechin-6-C-β-D-glucopyranoside
Flavanomarein
Vicenin 2
Sulfurein
Oroxin A
Homoplantaginin

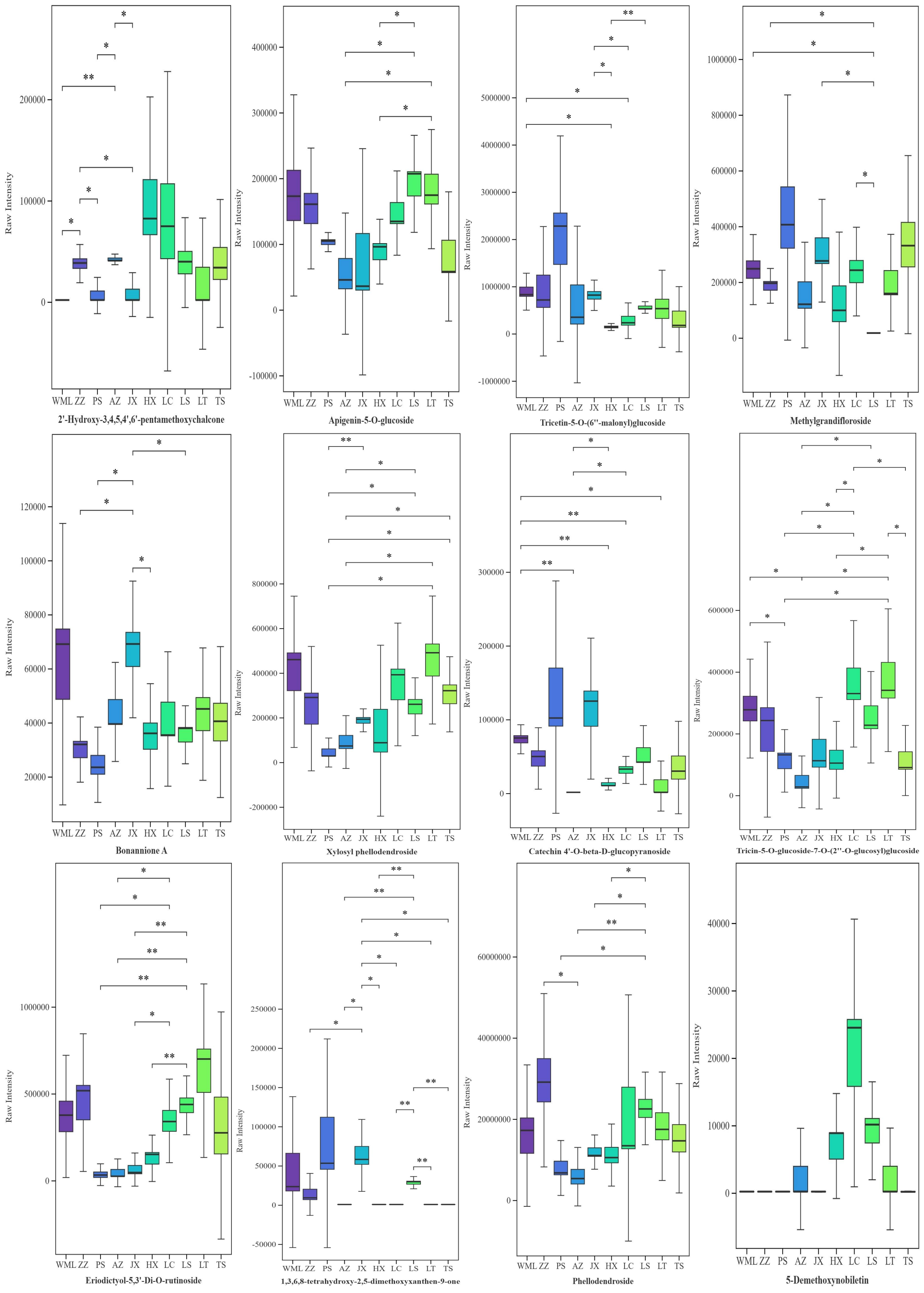

Raw Intensity
WML ZZ PS AZ JX HX LC LS LT TS
2'-Hydroxy-3,4,5,4',6'-pentamethoxychalcone
Apigenin-5-O-glucoside
Tricetin-5-O-(6''-malonyl)glucoside
Methylgrandifloroside
Bonannione A
Xylosyl phellodendroside
Catechin 4'-O-beta-D-glucopyranoside
Tricin-5-O-glucoside-7-O-(2''-O-glucosyl)glucoside
Eriodictyol-5,3'-Di-O-rutinoside
1,3,6,8-tetrahydroxy-2,5-dimethoxyxanthen-9-one
Phellodendroside
5-Demethoxynobiletin

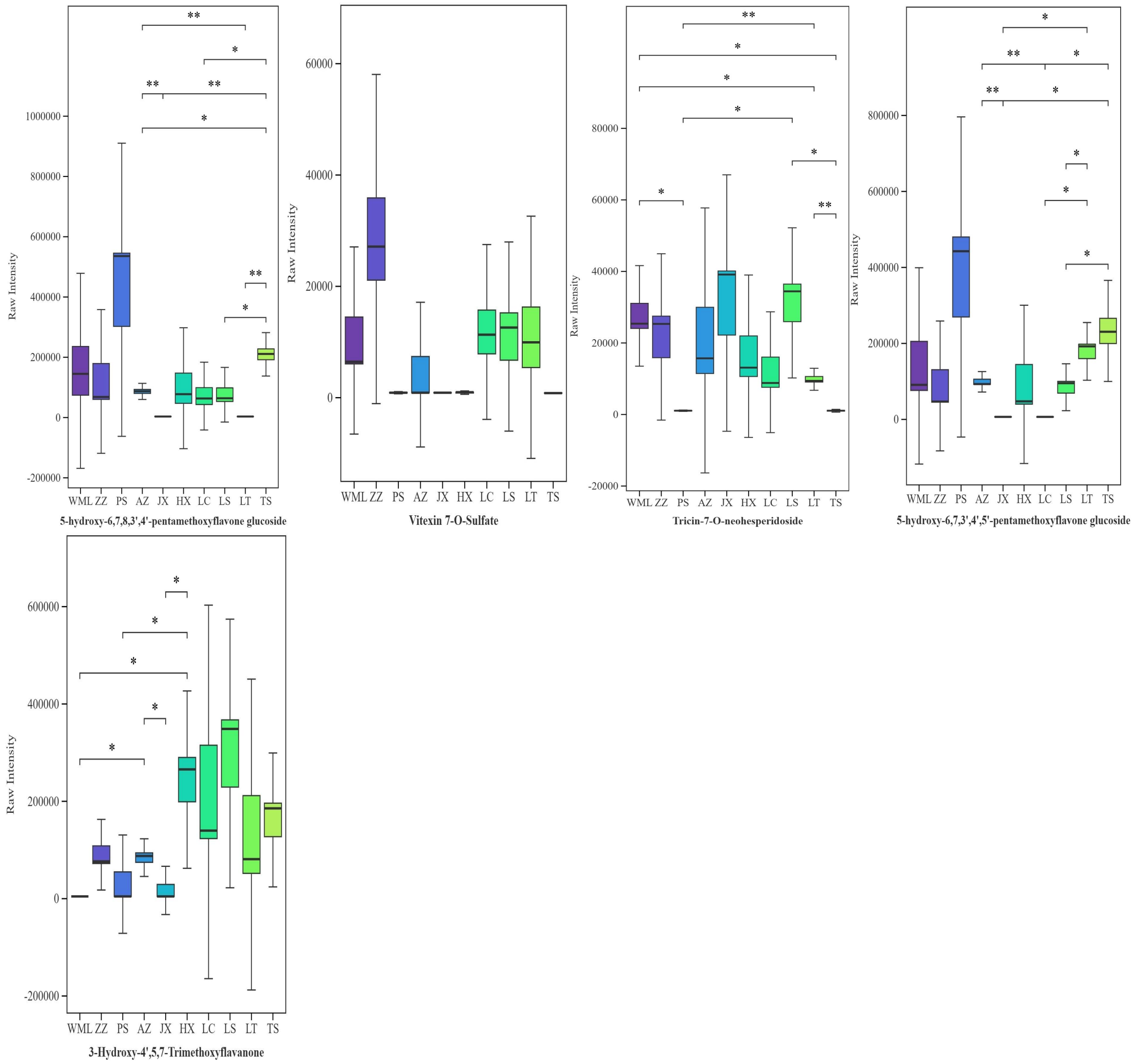

Figure S2 Advanced significance box plot of 29 flavonoid metabolites in *F. suspensa*.

Asterisks indicated significant differences according to Student's t-test (*, $p < 0.05$; **, $p < 0.01$; ***, $p < 0.001$; ****, $p < 0.0001$).
